# Body composition estimates from bioelectrical impedance and its association with cardiovascular risk

**DOI:** 10.4102/phcfm.v16i1.4587

**Published:** 2024-10-09

**Authors:** Jesne Kistan, Jeffrey Wing, Khanyisile Tshabalala, Wesley van Hougenhouck-Tulleken, Debashis Basu

**Affiliations:** 1 Department of Public Health Medicine, Faculty of Health Sciences, University of Pretoria, Pretoria, South Africa; 2 Department of Internal Medicine, Wits Health Consortium, Johannesburg, South Africa; 3 Steve Biko Academic Hospital, Pretoria, South Africa; 4 Department of Nephrology, Faculty of Medicine, Sefako Makgatho Health Sciences University, Pretoria, South Africa

**Keywords:** body composition, bioelectrical impedance, cardiovascular risk, South Africa, Primary health care

## Abstract

**Background:**

Screening for traditional risk factors of cardiovascular disease is well known in primary healthcare (PHC) settings. However, other risk factors through newer tools (such as bioelectrical impedance analysis [BIA]) could also be predictors of increased cardiovascular risk (CVR). Body composition estimates (body fat percentage, body water percentage, body lean mass) by BIA and its association to CVR have been studied with variable results.

**Aim:**

This study assesses the body composition estimates and their association with CVR in the South African PHC setting.

**Methods:**

A retrospective record analysis was conducted on a cohort of de-identified patients utilising the ABBY^®^ Health Check Machine at a PHC facility in South Africa between May 2020 and August 2022. The ABBY Machine estimates body fat percentage (BF%) and body water percentage (BW%) estimates from BIA. Cardiovascular risk based on the Framingham-risk-score was stratified into high, medium and low CVR. An analysis of variance was used to determine mean differences of BF% and BW% among these groups.

**Results:**

A total of 4008 records (*n* = 4008) were used in the final analysis. The majority of patients were female (70.1%) with a mean age of 33.6 years. Higher mean BF% (35.75% vs. 31.10% vs. 27.73%; *p* < 0.0001) and lower mean BW% (49.46% vs. 53.15% vs. 56.18%; *p* = 0000) were found to be significantly associated with high CVR.

**Lessons Learnt:**

This study demonstrated the use of newer technologies that could assist in the identification of CVR in low resource PHC settings.

## Introduction

Cardiovascular diseases (CVDs) including ischaemic, congestive, and hypertensive heart diseases form part of the top 10 causes of mortality in South Africa.^1^ Traditional risk factors for CVDs include modifiable (hypertension, diabetes, obesity, hypercholesteraemia) and non-modifiable (age, gender) factors. Prevention and control of the modifiable risk factors are central to preventing the development of cardiovascular risk (CVR).^2^

Identification of risk factors through low-cost, non-invasive instruments may be useful for the early prevention of CVDs. Bioelectrical impedance (BEI) instruments are low-cost, non-invasive tools^3^ that measure differential electrical conductivity through body tissues for calculation of body-fat, total water and lean-mass percentage based on bioelectrical impedance analysis (BIA). Although dual-energy X-ray absorptiometry (DEXA) machines are the gold standard for measuring body fat, there are practical and economic problems, such as expensive equipment requiring highly skilled personnel.^4^ With the advent of newer technology, contemporary equipment for BIA demonstrated a high correlation in body fat rate, body fat amount, and fat-free mass amount between DEXA and BIA devices. One such machine is the ABBY^®^ Health-check-machine, used at pilot sites in South Africa. ABBY-machines provide real-time risk screening for hypertension and CVR. Additionally, ABBY-machines collect patient demographics, history of chronic diseases, smoking, and measure weight, height, body fat, blood pressure, pulse-rate and oxygen saturation of an individual.^5^

Subsequently, it calculates body mass index (BMI), body fat percentage (BF%) and body water percentage (BW%) and estimates CVR based on Framingham Risk Score (FRS) independent of serum cholesterol and high- density lipoprotein values to calculate the FRS. The FRS is a composite score for the identification of patients at high risk of CVDs to offer appropriate preventive treatment.^6^ The BF% and BW% currently do not form part of the criteria used in the FRS. Numerous studies in Europe found an association between BEI-based body composition analysis and CVR.^7,8,9^ However, few studies were performed in South Africa exploring the relationship between body composition analysis and CVR. This study was planned against this background to investigate the association of BEI-based body composition measurement and CVR at primary healthcare (PHC) setting in South Africa.

## Methods

This was a cross-sectional study involving a retrospective record review of patients (*n* = 4008), who attended a PHC facility and used an ABBY-machine from May 2020 to August 2022. All adult patients (≥ 18 years), who attended that facility during the study period, were introduced to the ABBY-machine at the time of registration. Furthermore, the patients, who were willing to use the ABBY-machine, were introduced to it. The patients who made use of the machine were reincluded in the study. ABBY machine relies on BEI, which is a technique using electrical resistance to determine percentages of fat mass and fat free mass.

The following data were collected electronically by an ABBY-machine: patient demographics (age, sex, chronic disease history and smoking history), biometrics (weight, height, BMI, BF%, blood-pressure [BP], pulse-rate and oxygen-saturation) and composite measurements (BMI and FRS). Cardiovascular risk, according to the office-based FRS, was defined as low-risk (0% – < 3%), medium-risk (3% – 15%) and high-risk (> 15%).^10^ De-identified data (without patients’ name, email address and mobile number) were downloaded from the ABBY-machine and cleaned for any discrepancies and then analysed using STATA®13.^11^ Descriptive statistics were used to present normally distributed data using mean and standard deviations (s.d.). Otherwise, median, inter quartile range (IQR) were used. Comparison among the three groups (low, medium, and high cardiovascular risk) was performed using one-way analysis of variance. Post hoc test was used if test statistics were significant.

Permission for use of the de-identified clinical dataset was obtained from the owners of the ABBY Health Check instrument. All methods were undertaken in accordance with the regulations and guidelines set out by the South African Health Products Regulatory Authority (SAHPRA), the regulatory body for health products in South Africa.

### Ethical considerations

Ethical clearance to conduct this study was obtained from the University of Pretoria Faculty of Health Sciences Research Ethics Committee (No. 567/2021).

## Results

The demographic and clinical details of patients at their first visit attending the primary healthcare were presented in Table 1. The numbers of females and males in the study cohort were 2810 (70%) and 1198 (30%), respectively. Their mean age was 33.6 (±10) years, with no significant difference between female (33.0 ± 10 years) and male (34.1 ± 10.8 years) participants. The mean systolic (127 mm Hg ± 19 mm Hg) and diastolic (72.4 mm Hg ± 9 mm Hg) blood pressures, pulse rates (89 ± 19 per min) and oxygen saturation (98% ± 2%) were within the normal range. Their mean weight was 74.8 kg ± 17.2 kg. The majority of them were either overweight (1249, 32%) or obese (1101, 27%), and a third of them were normal weight and a few (3%) were underweight. Table 2 lists patients stratified by CVR as determined by their FRS. In the cohort with the highest CVR (FRS > 15%), the majority of the patients were male, aged 55 years, diabetic and had a higher BF% and lower BW% than patients with low or medium CVR. Using analysis of variance testing of the three groups, a higher mean BF% (35.75% vs. 31.10% vs. 27.73%) and lower mean BW% (49.46% vs. 53.15% vs. 56.18%) were significantly associated with higher CVR (*p* < 0.0001). Pairwise comparisons using the Tukey post-hoc test showed a statistically significant difference of body composition between high-risk versus low-risk (*p* < 0.001), medium-risk versus low-risk (*p* < 0.0001) and high-risk versus medium-risk (*p* < 0.0001). In the univariate model, both BW% and BF% were significantly associated with CVR (*p* < 0.001) (Table 3).

**TABLE 1:** Table showing the demographic and clinical details of patients at their first visit attending the primary healthcare.†

Variable	*N*	%	Mean	s.d.	Range	Median	IQR
Age (years)	4008	100	33.6	10.4	18.0–100.0	32.0	26.0; 39.0
**Body fat (%)**	**3279**	**81.8**	**29.2**	**10.8**	**2.8–71.6**	**28.5**	**21.5; 36.1**
Body fat (%) (female)	2268	69.2	33.3	9.4	26.4–38.8	32.1	12.2; 71.6
Body fat (%) (male)	1011	30.8	20.1	7.9	14.3–24.7	18.7	2.8; 62.2
**Body water %**	**4008**	**100**	**55.7**	**10.8**	**20.8–73.0**	**54.6**	**48.2; 62.5**
Body water (%) (female)	2810	70.1	53.5	11.3	20.8–73.0	51.7	46.2; 57.6
Body water (%) (male)	1198	29.9	60.7	7.5	27.9–73.0	60.6	56.1; 64.4
BMI (rr: 20.0 kg.m^−2^ – 24.9 kg.m^−2^)	3934	98.2	27.9	7.7	13.8–131.7	26.5	22.6; 31.7
Systolic BP (rr: 100 mmHg – 120 mmHg)	3727	93.0	127.3	19.2	62.0–226.0	126.0	114.0; 137.0
Diastolic BP (rr: 60 mmHg – 80 mmHg)	3727	93.0	72.4	9.3	45.0–129.0	72.0	66.0; 78.0
Weight (kg)	4008	100	74.8	17.2	34.4–168.6	72.4	62.0; 84.9
Height (cm)	3933	98.2	164.4	10.0	100.0–199.0	164.0	158.0; 171.0
Smoking	569	14.2	-	-	-	-	-
Diabetes	139	3.5	-	-	-	-	-
Framingham risk score	3727	93.0	4.3	7.1	0.1–84.6	1.8	0.9; 4.5

Note: Low risk < 3%; medium risk 3% – 15%; high risk > 15%.

s.d., standard deviation; IQR, interquartile range; kg, kilogram; cm, centimetre; rr, reference range; mmHg, millimetres of mercury; bpm, beats per minutes; kg.m^−2^, kilogram per square metre; FRS, Framingham risk score; BMI, body mass index.

† , Demographic and clinical information of patients in the final analysis.

**TABLE 2:** Demographic and clinical information of patients stratified by Framingham Risk Score using analysis of variance (ANOVA).

Variable	Low risk (0% – 3%)				Medium (> 3% – 15%)				High (> 15%)				*P*
	*n*	%	Mean	s.d.	*n*	%	Mean	s.d.	*n*	%	Mean	s.d.	
Female	1940	79.93	-	-	549	51.12	-	-	98	43.36	-	-	< 0.0001
Male	487	20.07	-	-	525	48.88	-	-	128	56.64	-	-	-
Age (years)	-	-	28.25	5.88	-	-	41.27	7.30	-	-	55.96	10.11	< 0.0001
Body fat (%)	-	-	27.73	9.39	-	-	31.10	12.24		-	35.75	13.15	< 0.0001
Body water (%)	-	-	56.18	9.71	-	-	53.15	11.10	-	-	49.46	11.84	< 0.0001
BMI	-	-	26.41	5.89	-	-	29.60	7.41	-	-	32.52	8.66	< 0.0001
Smoking (Yes)	230	9.48	-	-	253	23.56	-	-	58	25.66	-	-	< 0.0001
Diabetes (Yes)	9	0.37	-	-	47	4.38	-	-	74	32.74	-	-	< 0.0001

s.d., standard deviation; BMI, body mass index.

**TABLE 3:** Multivariate analysis of body composition elements and its associated cardiovascular risk.

Variable	Co-efficient (exp)	95% Confidence interval	*P*
Body water (%)	0.89	0.87–0.91	< 0.0001*
Body fat (%)	1.14	1.11–1.67	< 0.0001*

Co-efficient (exp), exponentiated regression coefficient.

* , statistically significant.

## Discussion

Bioelectrical impedance analysis could be suitable for epidemiological studies, surveys and clinical use for non-invasive measurement of body composition and screening for CVR in low- to middle-income countries (LMICs), where laboratory tests (such as serum cholesterol) are not readily available. Our study demonstrated that individuals with higher BF% and lower BW% had an increased CVR similar to other European studies.^7,8^ Our study adds to the body of literature,^12,13,14^ which supports association between body composition estimates and CVR.

Our study found association of lower BW% with higher CVR. It is known that hydration status affects the BF% and affects BEI measurements.^15^ However, there is minimal evidence explaining how hypo-hydrated states may predispose individuals to adverse cardiovascular events. There is evidence that acute hypo-hydration impairs vascular function and blood pressure regulation which could lead to cardiovascular events.^16,17^

Body fat percentage was found in some cases, as a better indicator of CVR than BMI.^18^ This could be as a result of some individuals with a normal BMI still having a higher BF%.^19^ While our study did not compare the predictive value of BF% versus BMI, we were able to show BF% to be significantly associated with increased CVR. Further consideration is needed to assess the predictive values of all body composition parameters and CVR in PHC settings.

We showed significant differences in age and gender in the low, medium and high CVR groups. However, as these factors form part of the FRS (collinearity), these factors could not be used in a multivariate-analysis. The study is not based on random sample and based on data collected from one primary health clinic, and therefore, its findings may not be generalised. However, this study is the first study in a PHC setting in South Africa, and a prospective longitudinal cohort study is needed to determine the relationship between body composition estimates and cardiovascular outcomes utilising BEI. Lastly, the study did not look into other factors (race, presence of chronic kidney disease, causes of dehydration such as diarrhoea), which might have an influence on CVR. Furthermore, a prospective longitudinal cohort study is needed to determine the relationship between body composition estimates and cardiovascular outcomes based on BIA.

## Conclusion

Our study demonstrated the use of body composition parameters other than BMI for measurement of CVR. Bioelectrical impedance analysis can be rapidly and conveniently used as a non-invasive tool, where laboratory tests are not readily available to quantify CVR in a PHC setting.
